# Reimagining a justice oriented professionalism: a call for relational ethics in oral health education

**DOI:** 10.3389/fdmed.2026.1819807

**Published:** 2026-07-17

**Authors:** Carlos S. Smith

**Affiliations:** Department of Dental Public Health and Policy, Virginia Commonwealth University, Richmond, VA, United States

**Keywords:** dental ethics, equity healthcare, ethical decision making (EDM), ethics, healthcare ethics, justice in health care, professionalism, relational ethics and care

## Abstract

Across the delivery of oral health care, whether in the halls of learning environments or the operatories of individualized clinicians, corporations, or even unverifiable private equity led acquisitions, ethics is largely seen as a theoretical exercise separate from the day-to-day realities of clinical practice. A call for relational ethics, not merely traditions' ethical principlism, is more than theoretical exercise or an intellectual inquiry. This call is quite literally coming from both inside and outside of the proverbial oral health house. In examining the absence of holistic community-centered commitments of principles such as veracity and justice, this article provides a framework for conceptualizing a reimagined professionalism across various domains of oral health care delivery. Anchored by truth-telling and anti-oppressive justice, the author explores educational practice and pathways, models of care delivery, barriers to optimal learning and clinical environments, while providing individualized and systemic recommendations for change, While the goals are robust, [re]building trust and shifting dentistry and oral health toward outcomes advancing real health equity is imperative for this hour. True ethical dental education and practice must move beyond individual behavior of patients and providers alike, toward systems of accountability, truth-telling, and community engaged partnership.

## 1 Introduction: the limits of traditional dental ethics

Traditional dental ethics has at its foundation the construct of principlism, an approach to ethical decision-making relying on a set of moral principles or standards to guide professional practice and behavior. Closely associated but distinct from codes of ethics, principlism provides the ethical theory and philosophical framework for ethical decision-making (1, 2). Codes of ethics can be thought of as expanding from theory to rules of engagement providing specific obligations, standards and even prohibitions. Often developed among professional associations broadly and organized dentistry specifically, codes of ethics in dentistry date back centuries to 1866 (3). That initial convening developed a robust code of ethics, while not expressly articulating principles as understood today, it instructed dentists to treat patients with honesty, care, and professionalism acknowledging that patients themselves may not be able to evaluate the quality of their treatment. It also required dentists to uphold the profession's honor by avoiding unethical advertising, respecting colleagues, and working responsibly with physicians and the public.

Although principle language was introduced as early as 1944 by the American Dental Association (ADA), it was not until the year 2000 that the ADA expressly implores bioethical and medical ethics principles of autonomy, beneficence, nonmaleficence and justice (4, 5). Interestingly the ADA explicitly names veracity, or truthfulness as its own stand-alone pillar while Beauchamp and Childress chose to frame truth-telling as a derivative duty flowing from respect for autonomy as patients need truthful information to make informed decisions (6). Veracity was most likely included as a foundational dental ethical obligation because dentistry has always been marked by the fact that dentists as clinician and expert possess greater, more detailed, or more accurate knowledge than the patient. In economics this phenomenon is noted as information asymmetries, and dentistry with its long history of public vulnerability to “quackery,” misleading claims, and professional exploitation rightly demands of itself to be a truth-teller. Even as far back as the 1873 Code the notion of truth-telling among dentists functioned as a necessary safeguard to protect patients who were “unable to correctly estimate the character of dental operations” (7).

While principlism obviously has its usefulness, there often remains a persistent gap between what some may deem as ethical theory vs. clinical realities (8–10). To be clear, ethics treated as abstract, peripheral, or “extra” rather than embedded in daily practice is unacceptable and counter to clinician professional duty. However, the disconnect between principlism (autonomy, beneficence, nonmaleficence, justice, veracity) and lived experiences of patients and providers continues to widen considering the continually evolving modalities of care delivery such as corporate dentistry, dental service organizations (DSOs), and private equity acquisitions (11). While production and revenue pressures have been a mainstay across oral health care delivery for decades, the changing landscape has reshaped the delivery of oral health care into high-volume, efficiency-driven systems where production quotas, cost-containment constraints, opaque decision-making hierarchies, advancing technology and social media savvy (or lack thereof) and unintended outcomes frequently put ethical commitments under duress (12). These structural tensions not only complicate ethical practice, they can fully distort it, creating environments where clinicians must negotiate tensions between what they know to be right and what the larger system demands of them. The full spectrum of oral health education, increasingly intertwined with these same pressures, mirrors these contradictions. Students learn principlism in the classroom while observing and participating in clinics where the subtle and not-so-subtle ways of revenue priorities override relational, patient and community-centered care. In this context, the limits of traditional ethics become plainly visible. The goal of this paper is to aid in presenting a true turnaround toward relational ethics. Defining relational ethics as an approach of oral health practice that understands ethics as lived, embodied, and co-created in the encounters between providers, patients, communities, and institutions. Relational ethics shifts the focus from minimal compliance with abstract principles to accountability within relationships, foregrounding mutuality, trust, and the moral significance of context. This reorientation and reimagining aligns with scholars calling for an ethics rooted in justice, community, and truth-telling. Bringing to life an ethics recognizing professionalism as not merely a set of rules or checklists but a shared commitment to the flourishing of all who participate in the oral health ecosystem.

## 2 Why principlism is not enough

In many ways principlism as oral health's ethical north star has been a much needed foundation, however the application of said principles to evolving oral health care delivery broadly and the continued exacerbation of health disparities and inequities, reveal a stark absence of actualized community-centered commitments (13). Relational ethics at its core focuses on the role of relational context or the experience of relationships in influencing moral choices (14, 15). Oral health's ethical codes tend to elevate individual decision-making while relegating veracity and justice to secondary or optional concerns, as though truth-telling and equity were mere add-ons rather than core obligations (16). This narrow framing allows systemic inequities to remain unexamined: the structural racism embedded in licensure pathways, the uneven distribution of providers, and the economic and political realities that shape who receives care and under what conditions (17, 18). Scholars have also shown the historic use of principlism and its focus on the individual clinician obscures the collective moral responsibilities of institutions, communities, and systems (19, 20). The systems orientation and lack of larger organizational ethics and responsibilities cannot be captured by the application of principles alone. When ethical guidance is reduced to personal virtue and rule compliance, the broader oral health professions more easily lose sight of the social contract that binds oral health to community well-being. To be clear, principlism is not wrong, it is simply insufficient for a world in which oral health disparities are produced not only by individual failings but by structural domains, and some might say intentionality, that all demand true ethical scrutiny and analysis.

Compounding this insufficiency is the persistent myth of neutrality that undergirds traditional dental professionalism (21). Oral health's supposed professionalism norms were historically defined through white, middle-class expectations of comportment, communication, and even the notion of “fit” (11). These standards have long been inappropriately used to police identity, behavior, and belonging. What counts as “professional” has too often been a proxy for assimilation, reinforcing hierarchies that marginalize students and providers of color and delegitimize the cultural knowledge they bring (22). The hidden curriculum in dental education is well established (23, 24). Those unwritten, unofficial, and often unintended lessons, values, and perspectives that students learn through institutional culture, various interactions, and clinical experiences. While not often discussed, these experiences shape ethical dispositions as well. Perhaps far more powerfully than any formal lecture on autonomy, beneficence, or other principles; it teaches students which patients are “difficult,” which colleagues are “unprofessional,” and which forms of advocacy are deemed “too political.” The consequences of this narrow ethical lens are paramount where the erosion of trust in communities that have long experienced dentistry as exclusionary. This is an inadequate preparation for the sometimes messy, relational dilemmas that define real-world practice, coupled with the reinforcement of care models that privilege efficiency over equity. Oral health is inseparable from the social and political conditions in which it is delivered (25). A professionalism that ignores these conditions cannot claim to be ethical, moreover it then only reproduces the inequities it refuses to name. Decades of scholarship have framed the fact that principlism based ethical decision-making and professionalism may be indeed insufficient (26–30).

## 3 Relational ethics as a framework for reimagined professionalism

If principlism is insufficient, where should oral health care turn for reimagining a justice-oriented professionalism? Relational ethics has its roots among the practice of one of society's repeatedly ranked most trusted professions, nursing. Nursing has been clear in naming at the most basic form, relational ethics helps to examine the space between people (healthcare providers and patients), their interactions and relationships, as the place where moral decisions, or ethical decision making, actually happens. Ethical discussion centers on the type and caliber of clinician connections with everyone who either directly or indirectly influences their profession. Nursing theory scholars have noted, “we can only live well autonomously if we live well together” (15). Further encapsulating the importance of relationships and the idea that moral goodness and happiness are derived from our interactions with others. Just as nursing's relational ethics insists that autonomy is only meaningful within networks of care, oral health professionalism must recognize that its highest ethical commitments are realized through relationships that honor interdependence, context, and justice (31).

With dentistry's own expressly including veracity as an aspect of its code of ethics, the jump to relational ethics as a framework for ethical analysis and care delivery is really no jump at all, but a truly logical and sequential next step. While this paper chooses to intentionally frame veracity in its full meaning as truth-telling, relational ethics brings this to life as an ongoing practice, not as a static or fixed principle. In fact, the pull away from principlism is also a nod to the dynamic nature needed for ethical framing for a continually evolving world, particularly related to oral healthcare delivery. Relational ethics allows for a delivery of oral health care via transparency, humility, and a willingness to confront histories of harm. Relational ethics allows for truth-telling as a moral encounter rather than a procedural requirement or checklist item (32, 33). Clinical relationships can be enhanced by shared meaning-making and truth-telling must include acknowledging the structural conditions that shape oral health inequities and the profession's complicity in them. This orientation reframes justice as an anti-oppressive obligation rather than an abstract ideal: a commitment to naming racism, classism, sexism, and other forms of structural violence as ethical concerns. For the purposes of this paper, *Anti-oppressive justice* frames ethical practice as actively resisting inequity and dismantling structural harms rather than merely ensuring distributive fairness. It calls clinicians and educators to recognize how racism, classism, and other systemic forces shape oral health outcomes and professional culture. Scholarship reinforces this stance by arguing that health professionals have a responsibility to address the social and political determinants that produce inequitable outcomes, not merely respond to their downstream effects (16, 34). In this way, relational ethics demands mutual accountability among institutions, providers, and communities. This ultimately requires an accountability grounded in shared vulnerability, historical awareness, and a commitment to repair. This concept of relational accountability refers to an ethical obligation to honor commitments within a multitude of relationships. Relationships between clinician and patient, faculty and learner, institution and community; all promoting ethically and justice-centered practice through transparency, follow-through, and responsiveness.

A theoretical example of this work can be found in scholars' analysis of the long-lauded artifact of George Washington's denture, long an item centering professional intrigue and pride, but more recently the subject of critical analysis (35, 36). Scholars note, “every country has its own myth-making, and part of US oral health lore is this complete denture from the country's first president. The denture is problematic because it is possibly composed of teeth from enslaved African people. Unnamed African people are removed from history, and yet their teeth are national lore. As an object, the denture is not a mere artefact of history, but is celebrated to show a nation's founding father's connection to a profession. To celebrate the denture without appreciating these ethical dilemmas is to miss the importance of critically engaging history and context in both oral health practice and dental education” (35). Thus relational ethics becomes especially vivid when applied to the stories we choose to tell and the ones we leave out, limit or erase all together. While simply a historical artifact to some, the denture is not a neutral object but a site of moral responsibility. To celebrate it without acknowledging the violence, coercion, and dehumanization embedded in its creation is to ignore the ethical demands of history, correlating to the present. For oral health and dental education, a relational ethic would mean teaching students that ethical practice requires truth-telling about the profession's past, attentiveness to the lived experiences of those harmed, and a commitment to naming the structural forces that shaped, and continue to shape, oral health inequities.

Not simply staying in a theoretical or abstract space, relational ethics also has applications to clinical care. Again, this historic example could reiterate a call to cultivate practices grounded in accountability, context, and community engagement (37–39). More than simply laughing away a mistrustful patient's articulated anxiety or apprehension, relational ethics speaks right to it. Ethical formation must include learning to recognize how power operates in everyday interactions, as well moral meaning as co-created through relationships. Truly justice-oriented care requires confronting the afterlives of structural violence and understanding how historical trauma shapes present-day mistrust (40–42). When students and clinicians engage the story of Washington's denture through this lens, they learn that ethical practice is not merely about technical competence or principled reasoning but it is honoring the humanity of those whose stories have been marginalized. Building trust through transparency, and ensuring that the profession no longer replicates the harms it once ignored need be top priority. This is the work of a reimagined professionalism, one that understands history as an ethical teacher and relationships as the foundation of justice-centered oral health care.

## 4 Relational ethics through narrative as a reimagined model for care delivery

To be clear, often the ethical issue is not simply the clinical act but the historical, relational, and structural context in which the act occurs. Relational ethics helps learners and clinicians understand that oral health care is always embedded in stories or narrative. Some may be told, some may be silenced, relational ethics allows for recognizing that justice requires attending to those stories with humility, truth-telling, and shared accountability (43). The following clinical scenarios or narratives allow for practical application of relational ethics mirroring the George Washington denture example and bring forth how justice, trust, and community can be rebuilt, or further broken, chairside.

### 4.1 A patient with a history of extraction trauma declines recommended treatment

A Black elder presents with severe periodontal disease and a clear clinical indication for multiple extractions.

Following the traditional principlism approach frames the dilemma narrowly simply considering a) autonomy: the patient refuses extraction; b) beneficence/nonmaleficence: the clinician believes extraction is necessary to prevent infection; and c) justice: the clinician offers the same treatment recommendation they would offer anyone else. Here, the clinician's ethical task is most often interpreted as ensuring informed consent, explaining risks, and then documenting refusal. The encounter becomes a technical negotiation about treatment acceptance. Relational ethics offers an opportunity to reframe the dilemma entirely. Upon further inquiry, the patient shares that their grandmother had teeth forcibly extracted in a segregated charity clinic, and that they themselves experienced rough dental care with no anesthesia as a child. Their refusal is not “noncompliance” but a response shaped by historical trauma, racialized harm, and generational memory. Like medical history, these relational histories are not peripheral but are ethically constitutive. They shape trust, vulnerability, and the moral meaning of care.

A relational ethical response shifts the clinician's obligations and duty where:

- Truth-telling becomes contextual, acknowledging dentistry's history of racialized extraction practices.
- Justice becomes anti-oppressive, requiring the clinician to recognize how structural violence shapes present-day fear.
- Accountability becomes mutual, with the clinician responsible for repairing and not simply dismissing the relational breach.
- Care becomes co-created, with the patient's narrative guiding the pace, approach, and goals of treatment.

Instead of pushing extraction, the clinician might begin with noninvasive care, build trust over time, and co-develop a plan for eventual extraction that honors the patient's history and agency. The ethical success of the encounter is measured not by procedural completion but by relational repair. Trust then becomes the most ideally measured outcome.

### 4.2 A student questions a “routine” extraction in a teaching clinic

A dental student observes a faculty member recommending extraction for a low-income patient even though the tooth is restorable. The justification is framed as efficiency: “It's faster, cheaper, and the patient won't maintain the restoration anyway.”

Again, relying solely on principlism offers limited guidance here with no principle being explicitly violated, unless perhaps recognizing the violation of informed consent and autonomy. While health care professionals often face competing principles, a decision to extract could be rationalized away as beneficence or justice through a perspective of cost-effective care.

Relational ethics exposes the deeper ethical problem that the recommendation itself is shaped by structural forces such as production pressures, insurance limitations, and assumptions or stigma about marginalized patients. The patient's voice is minimized (and sometimes fully silenced) and their capacity for self-determination is pre-judged. The hidden curriculum teaches students that some bodies are less worthy of restorative effort. The larger ethical question could be framed as, what kind of relationship is being created between the profession and this patient? What histories and inequities are being reproduced? A relationally ethical response would require:

- Engaging the patient in a genuine conversation about their goals and preferences.
- Naming the structural constraints rather than hiding them.
- Teaching students that justice requires resisting default assumptions about who “deserves” comprehensive care.
- Encouraging faculty to model ethical transparency rather than expediency.

This approach transforms the encounter from a technical proficiency decision into a moment of ethical formation for both student and patient, as well as being self-evaluative for the faculty.

### 4.3 A patient and clinician language barrier

A Vietnamese elder arrives with her adult daughter, who interprets informally. The clinician, pressed for time, rushes through the consent process for periodontal surgery, relying on the daughter's translation. The patient nods politely but later expresses fear and confusion, ultimately missing the appointment.

Principlism treats this as a failure of communication or patient follow-through. But relational ethics recognizes that ethical meaning is co-constructed, and that the encounter failed because the relational conditions for understanding were never created. Relational ethics reframes the clinician's obligations: to ensure access to a trained interpreter; to recognize that nodding may reflect cultural norms of deference rather than comprehension; and to understand that trust is built through pace, presence, and relational attunement, not rapid information transfer (44–46). Marginalized patients often carry histories of dismissal in clinical spaces, making relational humility essential. The ethical task becomes creating a space where the patient's voice is centered, not filtered through institutional haste.

### 4.4 Production pressures in a high volume practice with challenging team culture

In a high-volume setting, production targets create intense pressure on clinicians and the entire care team, shaping daily interactions and ethical tensions among colleagues. Consider an associate dentist who has recently returned from maternity leave and is navigating the demands of pumping breast milk during busy clinic hours. Despite her needs, the fast-paced environment offers no protected time or private space for pumping.

The unspoken expectation here is that the associate should prioritize production and revenue above all else. Possible issues in this type of office or team environment include but aren't limited to: a) strained staff relationships among where colleagues may express frustration or impatience, b) viewing breaks as disruptions to efficiency, and c) facilitating feels for the nursing mother of isolation and lack of support. Principlism might frame this as a matter of workplace policy compliance or individual accommodations. However, relational ethics reveals a deeper ethical dilemma again rooted in core values such as respect, care, and mutual recognition among colleagues. Relational ethics calls for a culture of empathic support, shared responsibility, and advocacy for structural change across the team. Ethical care extends beyond patient interactions to include how staff members honor each other's needs and dignity, and much scholarship shows a team that values and centers team member wellbeing actually leads to better patient outcomes. The nursing mother's well-being and ability to provide care for her child are integral to the ethical climate of the clinic and thereby patients as well. A relationally ethical response and team culture might involve team discussions to create protected pumping times, designate private spaces, and foster a culture where such needs are normalized rather than marginalized. The ethical success lies not only in meeting production goals but in building trust, solidarity, and justice within the care team, recognizing that ethical care is relational at every level of the clinical environment.

## 5 An oral health relational ethics decision making model

**Figure 1:**
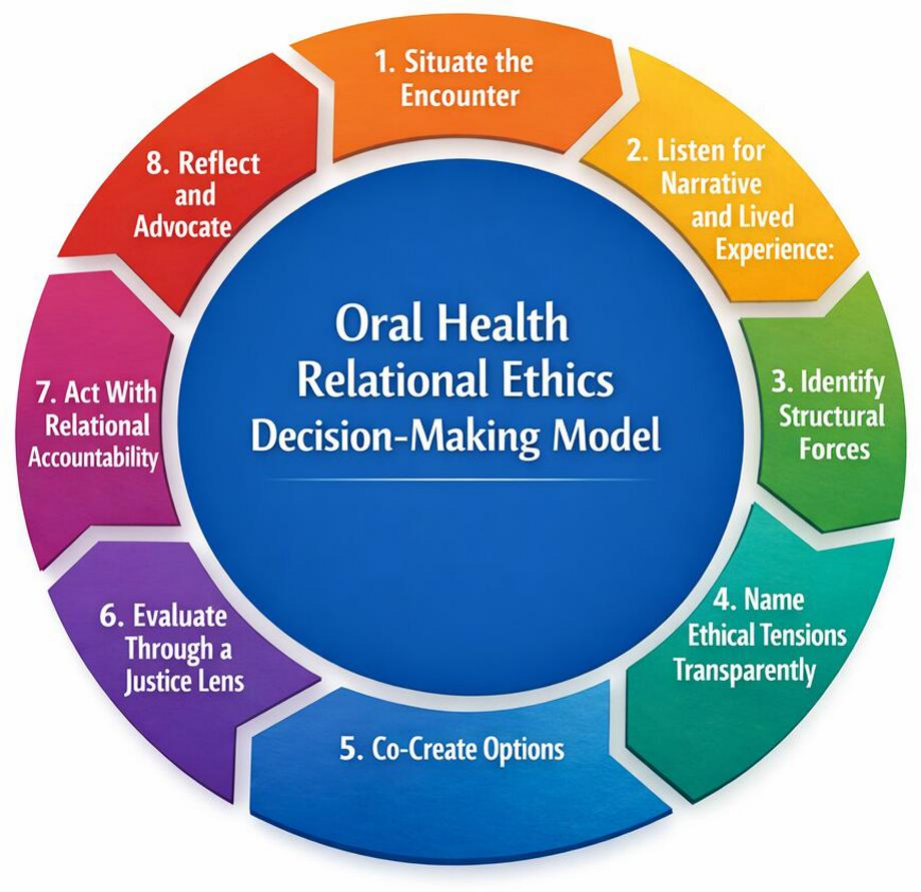
Oral health relational ethics decision making: An eight step circular model inserted here.

The Oral Health Relational Ethics Decision-Making Model is intentionally designed as a hybrid framework. See Figure 1. It aids in three primary ways: a) serving as an educational tool for ethical formation, b) as a reflective guide for clinical decision-making, and c) as a scaffold for institutional accountability. It is not prescriptive but interpretive, inviting learners and practitioners to use its steps as a lens for examining context, power, and relationship rather than as a checklist of compliance. A relational ethics decision-making model for oral health care must be and do more than sequence steps or offer check-lists. By both design and implementation it must shift the center of gravity from rule-based reasoning to context, history, power, and relationship. This paper offers a clear, teachable, clinic-ready model that learners and clinicians can use chairside.

### 5.1 Oral health relational ethics decision making: an eight step circular model

#### 5.1.1 Situate the encounter

Clinicians begin by grounding themselves in the context of the interaction. This includes recognizing power dynamics, historical trauma, and institutional forces that shape the moment. It prevents ethical reasoning from defaulting to abstract principles detached from lived experience.

Before identifying the “ethical issue,” clinicians can begin by asking:

What is the context of this encounter? What histories, across the personal, community, and institutional shape this moment? What power dynamics are present?

#### 5.1.2 Listen for Narrative and Lived Experience

This step centers the patient's story, fears, cultural meanings, and past experiences with dentistry or health systems. It reflects scholars' recognition of solidarity with patients and that marginalized patients' knowledge in particular must be treated as authoritative (11, 47, 48). It requires a full honoring of both embodied and historical knowledge (49, 50).

Instead of moving directly to options or consent, the clinician asks:

What is the patient's story of this condition or treatment? What fears, memories, or past harms shape their response? What community or cultural meanings are attached to this care?

#### 5.1.3 Identify Structural Forces

Clinicians examine how insurance limits, production pressures, inequitable policies, or the hidden curriculum may be influencing the encounter. Scholars work on political determinants of oral health underscores the importance of naming these forces (51, 52).

Clinicians must explore domains beyond simply the specified dental operatory or dental chair they find themselves in:

Are production pressures, insurance limits, or DSO policies shaping the recommendation? Are assumptions about “compliance,” “affordability,” or “appropriateness” influencing the plan? Is the hidden curriculum affecting how the patient is being perceived?

#### 5.1.4 Name Ethical Tensions Transparently

Rather than silently navigating constraints, clinicians articulate them openly. This truth-telling as practice is central to relational ethics and builds trust by acknowledging the realities shaping care.

Rather than silently navigating constraints by simply “keep their head down,” clinicians can outwardly articulate and process:

What are the competing obligations? What structural barriers should be acknowledged openly? What truths need to be told for trust to be possible?

#### 5.1.5 Co-Create Options

Instead of presenting predetermined choices, clinicians collaborate with patients to generate possibilities that honor their goals, histories, and constraints. This shifts from solely an informed consent lens to fully shared moral agency.

Instead of presenting pre-determined choices for patients, one can inquire:

What does the patient want for themselves? What alternatives can be created together? What timeline, pacing, or modifications honor their context?

#### 5.1.6 Evaluate Through a Justice Lens

Clinicians ask whether each option reinforces or resists inequity, whether it affirms dignity, and whether it builds trust. This step operationalizes anti-oppressive ethics, justice as anti-oppressive in practice and structural competency (16).

Justice compels that clinicians ask:

Does this option reinforce or resist inequity? Does it honor the patient's dignity, history, and community? Does it build or erode trust?

#### 5.1.7 Act With Relational Accountability

Ethical action includes follow-through, transparency, and responsiveness and not just performing a procedure. It reflects the full vision of professionalism as a relationship-centered accountability.

The clinician commits to:

Following through on what was promised, checking back in and adjusting the plan as trust deepens. Documenting not just the decision, but the relational reasoning behind it.

#### 5.1.8 Reflect and Advocate

Clinicians reflect on what they learned, how their assumptions shaped the encounter, and what structural changes require advocacy. This step ensures that ethical growth is iterative, harkens to dentistry's long held commitments to life-long learning, and aims to ensure individual encounters inform institutional transformation (53–55).

After the encounter:

What did I learn about this patient's world? What structural barriers need advocacy beyond this visit? How did my own assumptions shape the interaction?

This new model for ethical decision making can extend beyond theoretical framing into actual daily practice and pedagogy. Further clinical setting examples of step wise utility might include *Situate the Encounter* involving a provider pausing before treatment to acknowledge historical mistrust or community trauma shaping the patient's experience. *Co-Create Options* could appear when a clinician and patient collaboratively design a treatment plan that honors financial realities and cultural meaning. In educational contexts, faculty can model *Name Ethical Tensions Transparently* by openly discussing production pressures or institutional constraints during case conferences. Students may use the model in ethics or grand rounds to analyze how structural forces influence care decisions, thereby learning to integrate justice and narrative into their clinical reasoning.

The circular form of the model is intentional by design communicating that ethical decision making is continuous, relational, and recurrent. There is not necessarily a final ethical act, rather exhibiting and cultivating an ongoing commitment to justice, truth-telling, and community. It visually reinforces that relational ethics is not a checklist but a way of being in relationship with patients, colleagues, and communities.

While relational ethics offers a transformative lens, principlism remains foundational for articulating universal moral duties. The two frameworks are not adversarial but complementary: principlism provides normative anchors such as autonomy, beneficence, nonmaleficence, justice, and veracity. While relational ethics contextualizes their application within lived experience and structural realities. This coexistence ensures that oral health ethics remains both principled and responsive to the complexities of care delivery. In clinical decision-making, relational ethics and principlism operate dynamically. A clinician may begin with principlist reasoning such as respecting autonomy and beneficence, then expand through relational inquiry to situate those principles within the patient's narrative and structural context. The model thus functions as an interpretive extension rather than a replacement, enabling ethical reasoning that is both principled and relationally grounded.

## 6 Additional domains of oral health education requiring transformation

In short, relational ethics is a unique and impactful way for the profession to make significant inquiry and inroads of truly reimagining long held systems and standards that no longer hold up to their promise. Particularly amongst oral health, where individualized interventions have long been a mainstay vs. systems thinking or population health, the question often asked is what should we or what could we do? Relations ethics asks, the more rare inquiry of why the system is designed this way, how else could it be co-designed with all participants and parties in said system? Thus, relational ethics demands a transformation not only in clinical practice but further upstream to examine the entire dental education landscape including admissions, mentorship, curriculum and institutional culture (56). Current admissions metrics, such as the Dental Admission Test (DAT), remain dominant despite limited evidence of their predictive validity. While some studies suggest modest correlations between DAT scores and early academic performance, particularly in preclinical courses, others highlight significant limitations, including cultural bias and narrow cognitive scope (57–59). A relational ethics framework would shift admissions toward true holistic evaluation: valuing lived experience, community engagement, and relational aptitude alongside academic metrics (60–62). This would better identify candidates capable of co-creating trust and justice in clinical space and ultimately improving patient care that is culturally responsive. Similarly, curriculum design must move beyond mere technical competence to include structural competency, narrative ethics, and community-engaged learning. Assessment strategies should reward ethical reasoning that integrates context, history, and power and not just procedural recall. This is paramount when one considers the ethical lapses and regulatory breaches in future practice compared to learner status quo admissions or academic metrics. Studies have shown that professionalism lapses in medical school were associated with later disciplinary actions and problems in residency and clinical practice (63–66). Even more resolutely, academic strength alone is not protective, being that many sanctioned physicians had strong academic records; what distinguished them was not knowledge or test scores, but patterns of unprofessional behavior, poor moral reasoning, or problematic identity formation that were never adequately addressed. Mentorship, whether of learners or faculty, must be reimagined as a relational practice that fosters belonging, agency, and moral imagination, especially for students from historically excluded backgrounds (65–67).

Learning environments themselves must also be interrogated through a relational ethics lens (17, 68, 69). Psychological safety, often undermined by hidden curricula and professionalism norms that police identity, is essential for ethical formation. Faculty role modeling also plays a critical role. When educators recognize and name structural constraints, honor patient narratives, and practice relational accountability, they teach students that ethics is lived, not a mere abstract notion. Institutional culture must support this by valuing reflection, humility, and justice-oriented inquiry. Relational ethics also challenges the practice of sending the least trained learners into the most underserved communities, a model that left interrogated risks treating students as a cheap labor force and patients as merely training material. Instead, relational ethics would ask: What relationships are being built? What histories are being honored or ignored? Are students prepared to engage ethically with communities shaped by trauma, exclusion, and mistrust? When learners are supported to enter these spaces with humility, structural awareness, and relational accountability, their presence can be part of a justice-oriented care model and not an exploitative workaround. This requires institutions to invest in preparation, supervision, and community partnership and not just placement. Again, relational ethics becomes a guide not only for individual decisions but for systemic transformation.

## 7 Limitations

This manuscript advances relational ethics as a much-needed framework for reimagining professionalism in oral health education and practice; some limitations are noted. First, relational ethics itself is not a fully standardized or universally adopted model within dentistry, and its application across diverse institutional, cultural, and clinical contexts will require adaptation, ongoing evaluation, and empirical study. Second, this paper draws from interdisciplinary scholarship across nursing, bioethics, and structural theory; the translation of these frameworks into dental curricula and care systems remains uneven, and evidence on implementation outcomes is emerging. Third, the analysis relies on illustrative narratives and historical examples that, while grounded in documented patterns of inequity, cannot capture the full scope of patient experiences or institutional realities across the profession. Finally, structural transformation requires institutional will, resources, and cultural change that extend beyond the scope of any single framework; relational ethics offers a powerful orientation, but it must be paired with policy reform, faculty development, community partnership, and accountability mechanisms to achieve lasting impact. There must also be the intentionality and willingness for leadership across dental education and its institutions to move beyond mere status quo and risk aversion compliance to actualized commitment and transformation. These limitations underscore the need for continued research, dialogue, and collaborative efforts as the profession moves toward a justice-oriented future.

## 8 Future research

Future research is needed to examine and operationalize relational ethics across the full continuum of oral health education and care delivery. Empirical studies could examine how relational ethics informed curricula influence student success, ethical reasoning, and readiness to engage with structurally marginalized communities. Longitudinal research could explore whether graduates trained in relational ethics demonstrate different patterns of patient trust, treatment planning, and community engagement compared to those formed under traditional principlism. Additional inquiry is also needed into admissions practices, including the predictive validity and equity implications of the DAT, and whether relational competencies like cultural humility, structural awareness, and relational accountability can be reliably assessed in candidate selection. Research on learning environments has been established but must continue, particularly in the nation's current anti diversity, equity and inclusion environment. Future studies could evaluate the ethical and community impact of sending learners into high-need settings, assessing whether these models foster genuine partnership or inadvertently reproduce extractive labor dynamics. Community-engaged research can help clarify how patients and communities define ethical care, what relational practices build trust, and how institutions can be held accountable for historical and ongoing harms. Finally, implementation science approaches are needed to identify the policies, structures, and faculty development strategies that enable relational ethics to take root across diverse dental schools, DSOs and private equity owned practices, and care environments. Together, these research directions can help build an evidence base that moves relational ethics from conceptual aspiration to a measurable, transformative force in oral health education and practice.

## 9 Conclusion

A justice-oriented professionalism requires an embrace of relational ethics as dentistry's renewed moral center, not merely an option to principlism. Ethical transformation cannot be achieved through individual goodwill alone but demands structural change in how institutions teach, evaluate, mentor, and relate to the communities they serve. Dental education and practice carry a moral obligation and duty to confront the histories and power dynamics that shape oral health inequities, to cultivate learning environments where belonging and psychological safety are non-negotiable, and to form clinicians who understand that truth-telling, mutual accountability, and anti-oppressive justice are core professional duties. Moving toward this future requires institutions to reimagine admissions, redesign curricula, model relational integrity, and build community partnerships grounded in humility and collaboration rather than extraction. It calls educators to prepare learners not simply to perform procedures but to enter relationships with awareness, courage, and care. And it invites clinicians and communities to hold one another accountable for building an oral health system worthy of trust. The path forward is clear to a profession committed to justice, that must also commit to relationships. The ethical future of oral health depends on how well we learn to live, work, and truly care with one another.

## Data Availability

The original contributions presented in the study are included in the article/Supplementary Material, further inquiries can be directed to the corresponding author.
